# Gene expression supports a single origin of horns and antlers in hoofed mammals

**DOI:** 10.1038/s42003-024-06134-4

**Published:** 2024-05-20

**Authors:** Zachary T. Calamari, John J. Flynn

**Affiliations:** 1 Division of Paleontology, American Museum of Natural History, Central Park West at 79th Street, New York, NY 10024 USA; 2 Richard Gilder Graduate School, American Museum of Natural History, Central Park West at 79th Street, New York, NY 10024 USA; 3 Present Address: Department of Natural Sciences, Baruch College, City University of New York, 17 Lexington Avenue, Box A-920, New York, NY 10010 USA

**Keywords:** Evolutionary genetics, Gene expression, Evolutionary developmental biology

## Abstract

Horns, antlers, and other bony cranial appendages of even-toed hoofed mammals (ruminant artiodactyls) challenge traditional morphological homology assessments. Cranial appendages all share a permanent bone portion with family-specific integument coverings, but homology determination depends on whether the integument covering is an essential component or a secondary elaboration of each structure. To enhance morphological homology assessments, we tested whether juvenile cattle horn bud transcriptomes share homologous gene expression patterns with deer antlers relative to pig outgroup tissues, treating the integument covering as a secondary elaboration. We uncovered differentially expressed genes that support horn and antler homology, potentially distinguish them from non-cranial-appendage bone and other tissues, and highlight the importance of phylogenetic outgroups in homology assessments. Furthermore, we found differentially expressed genes that could support a shared cranial neural crest origin for horns and antlers and expression patterns that refine our understanding of the timing of horn and antler differentiation.

## Abstract (web summary)

An RNA sequencing study comparing juvenile cattle horn buds to growing deer antler tissues finds gene expression patterns that support homology of horns and antlers relative to a cranial-appendage-lacking outgroup.

## Introduction

**Fig. 1:**
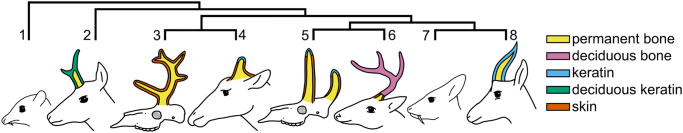
Cranial appendage tissue composition and phylogenetic relationships of living and extinct ruminants. Living and entirely extinct (†) ruminant families (even-toed hoofed mammals; Artiodactyla, Ruminantia), showing cranial appendage types and tissue composition. 1: Tragulidae, 2: Antilocapridae (pronghorns), 3: Climacoceratidae†, 4: Giraffidae (ossicones), 5: Palaeomerycidae†, 6: Cervidae (antlers), 7: Moschidae, 8: Bovidae (horns). Tragulidae and Moschidae lack cranial appendages. Phylogeny based on Hassanin et al.^17^.

Homology is difficult to establish for the horns, antlers, ossicones, and pronghorns (cranial appendages) of extant ruminants (Artiodactyla, Ruminantia), the clade of even-toed hoofed mammals (cattle, antelopes, deer, and relatives). Biologists use different names for the cranial appendages of each living ruminant family because their tissue composition and growth are distinct (Fig. 1). Nevertheless, all ruminant cranial appendages include permanent bony outgrowths of the frontal and parietal bones with family-specific integument coverings^1–3^. Although some researchers use these morphological and developmental differences to infer independent origins for the distinct types of cranial appendages^1^, both phylogenetic optimizations based on alternative concepts of cranial appendages and genomic data instead may support a single origin and deep homology of cranial appendages in ruminants^4–6^. Both interpretations are plausible, yet their implications for understanding ruminant craniofacial biology and morphological diversification are profound. In this study, we focus on the horns of Bovidae (e.g., antelopes, goats, cattle) and the antlers of Cervidae (e.g., moose, white-tailed deer, reindeer) to address the question of homology with high-throughput RNA sequencing analyses. These families have the greatest species diversity of living ruminants and include common livestock and game species, which provides better access to their cranial appendage tissues than is possible with the endangered Giraffidae (giraffes and okapi with ossicone appendages) and wild Antilocapridae (pronghorn “antelopes” with pronghorn appendages).

Bovid horn growth appears to begin in a specialized region of the dermis and subcutaneous loose connective tissue (SLCT) overlying the frontal bone of the skull, often called the *os cornu*^1^. Although inexact use of the term *os cornu* has confounded efforts to determine whether it is a separate ossification center^1,3^, recent examinations show substantial increases in nerve fibers and other morphological changes in developing horn bud dermis relative to other skin overlying the frontal bone^7^. The tissues clearly promote horn growth through intramembranous ossification^1^; however, they may not convert epidermis into the keratin sheath, suggesting horn bud and keratin sheath tissues receive these fates separately *in utero*^1,7–9^. Although the horn bud is distinct from frontal skin in fetal cattle by the second month of gestation, ossification of the bone core only occurs after birth^7^.

In contrast, the periosteum covering the frontal bone, not the more superficial SLCT and dermis, initiates antler growth^1,10,11^. During early growth of the pedicle, the permanent bone base of an antler, ossification is intramembranous; later pedicle growth and subsequent antler growth from the pedicle are both endochondral^1,11^. As with horns, the tissues that give rise to the antler receive this fate *in utero*, differentiating from surrounding tissues by the second month of gestation^11^. Unlike the early postnatal onset of horn growth in bovids, pedicle and antler growth in most cervids does not begin until puberty^11^.

Debate over the origins of cranial appendages involves two competing concepts of cranial appendages as anatomical structures. The first concept includes any family-specific bone or integument covering the permanent bony portion as an essential part of the structure. This “essential integument” concept emphasizes several differences between cranial appendage types to argue that each must have originated independently, such as the tissues that appear to initiate their growth, the type of ossification characterizing their main growth, and the timing of onset of growth. Under the essential integument concept, the differences in horn and antler development and integument covering become definitive indicators of non-homology—a horn cannot have shared origins with an antler, because no known fossil or living cervid has a keratin sheath on its cranial appendages and no known bovid has ever had a deciduous bone shed and regrown annually from its cranial appendage. This concept requires some ruminant families to have evolved cranial appendages multiple times. For example, the extinct merycodontine antilocaprids likely had skin covering their cranial appendages, while their extant relative, *Antilocapra americana*, has deciduous keratin sheaths covering its cranial appendages^1,12^. The essential integument concept treats these as two separate originations, solely because of the difference in their integument covering. The presence of a bony core in all antilocaprids, however, indicates that the group ancestrally possessed a bony cranial appendage through simple character optimization, and the evolution of a merycodontine cranial appendage into one like that of *A. americana* could have occurred simply through changes to the integument covering this bony core^12^.

Contrasting the essential integument concept is the hypothesis that each cranial appendage evolved by modification of a shared rudimentary ancestral cranial appendage. Under this concept, the permanent bone portion of each cranial appendage is a “homologous core” structure and was present as some rudimentary outgrowth in the common ancestor of all ruminants. The differences between each cranial appendage type, such as the permanent keratin sheath of a horn or the deciduous skin-covered bony antler, thus are independently evolved elaborations upon this common ancestral bony base^13^. The potentially separate fate determination of the horn bud and keratin sheath during embryonic development^7–9^ could be evidence that the keratin sheath evolved independently from the bony core. The similar position on the skull and initial stages of intramembranous ossification of both horns and antlers (as well as ossicones and pronghorns of other ruminants not included in this study) also would reflect a shared origin of cranial appendages from an ancestor with a rudimentary, intramembranously ossified bony frontal outgrowth. Recently discovered stem fossils from cranial appendage-bearing families evince rudimentary versions of their respective cranial appendages^14,15^. They demonstrate the potential for fossil discoveries to provide morphological support for the rudimentary ancestral cranial appendage of all ruminants proposed by this hypothesis. In the current paucity of such fossil evidence, shared and derived genetic mechanisms that initiate and direct cranial appendage growth in living ruminants can provide the best evidence for the homologous core concept.

Regardless of which cranial appendage concept applies, homology is fundamentally a question of phylogenetic optimization of traits, be they transcriptomic, genomic, or anatomical. Phylogenetic analyses of molecular data provide well-supported hypotheses of ruminant family interrelationships (Fig. 1) that prompt re-examination of cranial appendage homology^3^. Notably, these studies find cranial appendage-lacking Moschidae (musk deer) nested within cranial appendage-bearing ruminants as the closest relative to horn-bearing Bovidae^16–18^ rather than outside the living families with cranial appendages. Although both positions support a most parsimonious ancestral origin for cranial appendages in ruminants, a Moschidae + Bovidae clade introduces the possibility of more complicated scenarios of gains and losses. Likewise, molecular phylogenetic analyses place antler-lacking water deer *Hydropotes inermis* well within the antlered cervids as the closest relative to the antler-bearing roe deer genus *Capreolus*, rather than as the nearest outgroup to all antler-bearing cervids^17,19^, rejecting the possibility that this species supports an antlerless origin for Cervidae. Under the homologous core concept, the most parsimonious interpretation of antlerless *Hydropotes* would be a secondary loss of antlers regardless of where it falls in the cervid phylogeny, because the bony antler pedicle would be inherited from the common ancestor of all cranial appendage-bearing ruminants. Overall, these current phylogenetic positions of Moschidae and *Hydropotes* require only a single origin of cranial appendages within ruminants and separate losses in these two groups. Separate origins for each cranial appendage type, inclusive of its elaborations, based on the a priori adoption of the essential integument concept, instead requiring four or more gains or losses of cranial appendages across ruminants.

Just as the addition of molecular evidence has altered our understanding of ruminant evolutionary relationships, it also can enhance our understanding of cranial appendage homology. If cranial appendages first arose as a rudimentary bony structure in the ruminant common ancestor, shared biological processes may initiate and sustain their growth in all extant ruminants. These shared processes should be evident through the genes expressed during development^1,3,20–22^. High-throughput sequencing studies of horns or antlers, however, have focused on these structures within a single species or type of appendage^23,24^. The few studies of gene expression in cranial appendages that have compared different cranial appendage types provide valuable insights into genes that may support homology but lack the outgroup comparisons necessary for a rigorous, phylogeny-constrained test of homology^4–6^.

Additionally, the timing and rate of development of structures can evolve, and these changes are often responsible for evolving novel morphology^25^. For example, changing when tooth formation begins may result in teeth appearing to form from different layers of oral or dental epithelia, even though the teeth themselves are homologous structures^26^. A similar heterochronic shift may be responsible for developmental differences in timing and tissue layer observed between cranial appendages. Antler growth and regeneration are driven by stem cells in the periosteum^11^; although stem cells have not yet been identified in horn growth, formation of the *os cornu*, horn core, and keratin sheath likely involve some aspect of mesenchymal cell differentiation. In particular, both horn and antler precursors likely form from cranial neural crest cells^6,11,23,27^, although direct experimental confirmation is lacking. Regardless, developmental differences or similarities between two groups are not sufficient by themselves to reject or establish homology; they also must be considered within the context of the ancestral conditions and subsequent modifications reconstructed through phylogenetic outgroup comparisons.

In this study, we assessed the homology of cranial appendages under the homologous core concept, testing the hypothesis that the horn buds of juvenile cattle are homologous to antler pedicle tissues. We compared genes expressed by a developmental sequence of juvenile cattle horn buds to those expressed by deer antler pedicles, antler tips, and isolated antler tip tissues to explore heterochronic changes in expression timing that may support homology. We included a baseline outgroup comparison to pig skin, cartilage, and bone to assess homology. We predicted that the proportion of homologously expressed genes shared between horn buds and antler tissues would decrease over development, reflecting an evolutionarily conserved early trajectory for the permanent bone base that only differentiates later in development to yield family-specific features. Horn buds are essentially skin that can induce additional bone growth, so we expected a significant overlap with genes differentially expressed (DE) in the skin. Focusing on genes and pathways that differentiate cranial appendages from these within-species skin controls, we identified multiple genes with homologous expression patterns between cattle horns and deer antlers. Our results support the homology of horns and antlers through gene expression and highlight the need to include a phylogenetic outgroup in homology assessments using genomics data. Our results also may support cranial neural crest derivation of horns and suggest regulatory genes of interest for future analyses of cranial appendage evolution.

## Results

### Homologous differential expression patterns

**Fig. 2:**
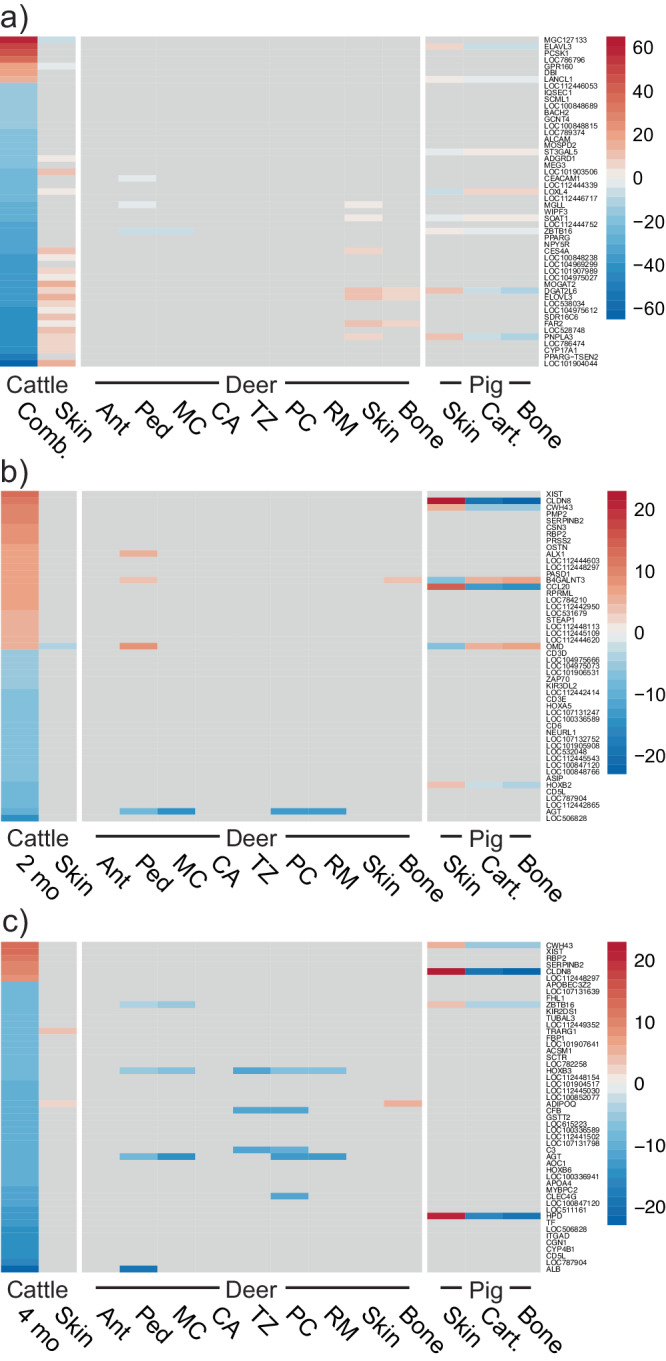
Top 50 differentially expressed genes for cattle horn buds. Heatmaps of log fold changes for the top 50 significantly differentially expressed genes in each cattle horn bud comparison, focusing on genes expressed in a different direction or not significant in cattle skin and including the log fold changes for the genes in the deer antler and pig tissues. Heat scales in each heatmap are log fold change. **a** Combined cattle horn bud analysis (2- and 4-month-old cattle horn buds tested together). **b** Two-month-old cattle horn bud analysis. **c** Four-month-old cattle horn bud analysis. Figure panels display combined cattle horn buds (Comb.), 2-month-old cattle horn bud (2 mo), 4-month-old cattle horn bud (4 mo), bulk deer antler tip (Ant), bulk deer antler pedicle (Ped), isolated deer antler mineralized cartilage (MC), isolated deer antler cartilage (CA), isolated deer antler transition zone (TZ), isolated deer antler pre-cartilage (PC), isolated deer antler reserve mesenchyme (RM), and control cartilage (Cart.), skin (Skin), and bone (Bone) unrelated to cranial appendages.

**Fig. 3:**
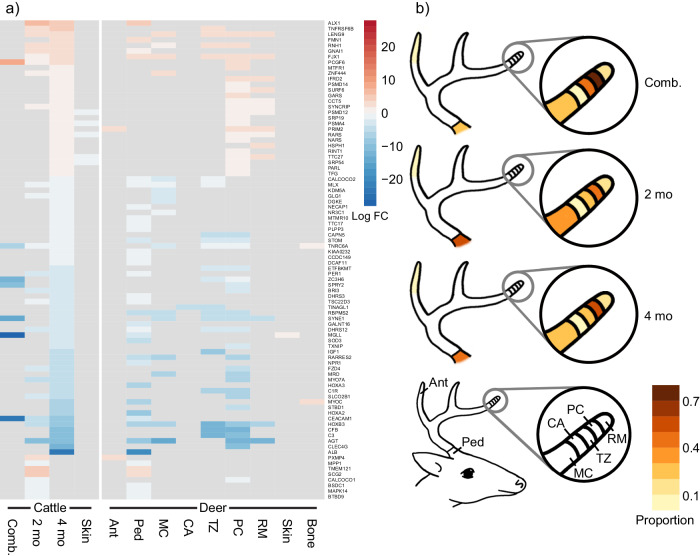
Homologous gene heatmap and shared proportions between cattle horn buds and deer antlers. **a** Heatmap of log fold changes (log FC) for the differentially expressed genes that mapped as homologous (expressed in the same direction in at least one bovid horn bud and cervid antler tissue and expressed in the opposite direction or not significant in pig tissues; genes expressed in the same direction as within-species skin or bone controls excluded). **b** Proportions of homologously expressed genes shared between each cattle horn bud analysis (combined, 2-month-old, and 4-month-old) and each deer antler tissue. Figure panels display combined cattle horn buds (Comb.), 2-month-old cattle horn bud (2 mo), 4-month-old cattle horn bud (4 mo), bulk deer antler tip (Ant), bulk deer antler pedicle (Ped), isolated deer antler mineralized cartilage (MC), isolated deer antler cartilage (CA), isolated deer antler transition zone (TZ), isolated deer antler pre-cartilage (PC), isolated deer antler reserve mesenchyme (RM), and skin (Skin) and bone (Bone) unrelated to cranial appendages.

We identified 270 significantly DE genes in the combined age cattle horn bud analysis (both 2- and 4-month-old horn buds analyzed together), 2717 significantly DE genes in the 2-month-old horn bud, and 5486 significantly DE genes in the 4-month-old horn bud (Fig. 2a–c; tables of all significantly DE genes for the combined and separate age analyses are in supplementary data 1 and 2). The combined age cattle horn buds and at least one deer antler tissue expressed seven genes homologously, i.e., the gene was expressed in the opposite direction or not significantly expressed in the outgroup tissues (Fig. 3a, supplementary data 3). Two-month-old horn buds and at least one antler tissue shared 30 homologously expressed genes, and 4-month-old horn buds expressed 77 genes homologously. The proportion of homologously expressed genes shared between the combined age cattle horn analysis and individual deer antler tissues did not follow a clear pattern across the samples by their relative ages (Fig. 3b). Two-month-old cattle horn buds shared most homologous genes with the bulk deer pedicle followed by isolated deer antler pre-cartilage. The 4-month-old horn bud had more homologously expressed genes in common with isolated antler pre-cartilage than with bulk antler pedicle.

Multiple genes were significantly DE in two or more of the horn bud analyses. *CEACAM1* appears in all three analyses, three genes were significantly DE in the combined age horn and 4-month-old analysis, and 22 genes were significantly DE in both 2-month-old and 4-month-old horn buds. Two homologous significantly DE genes appear to differentiate signaling for bone development in cranial appendages from that of unrelated bone, *TNRC6A* and *MYOC*. *TNRC6A* was underexpressed in all three horn bud analyses as well as antler-isolated mineralized cartilage, isolated transition zone, and isolated pre-cartilage, and *MYOC* was underexpressed in 4-month-old cattle horn buds and bulk deer antler pedicles. Both genes were overexpressed in the deer bone sample. Other potentially notable significantly DE genes in one or more horn bud sample, although not all expressed in a homologous pattern, include *ACAN, ALX1*, *CRABP1*, *DLX1*, *DLX2*, *ID*, *MAPK14, SOX9, SOX10, TFAP2A, TFAP2B*, and *TFAP2C*.

### Competitive gene set ranking and regulatory overlaps

**Fig. 4:**
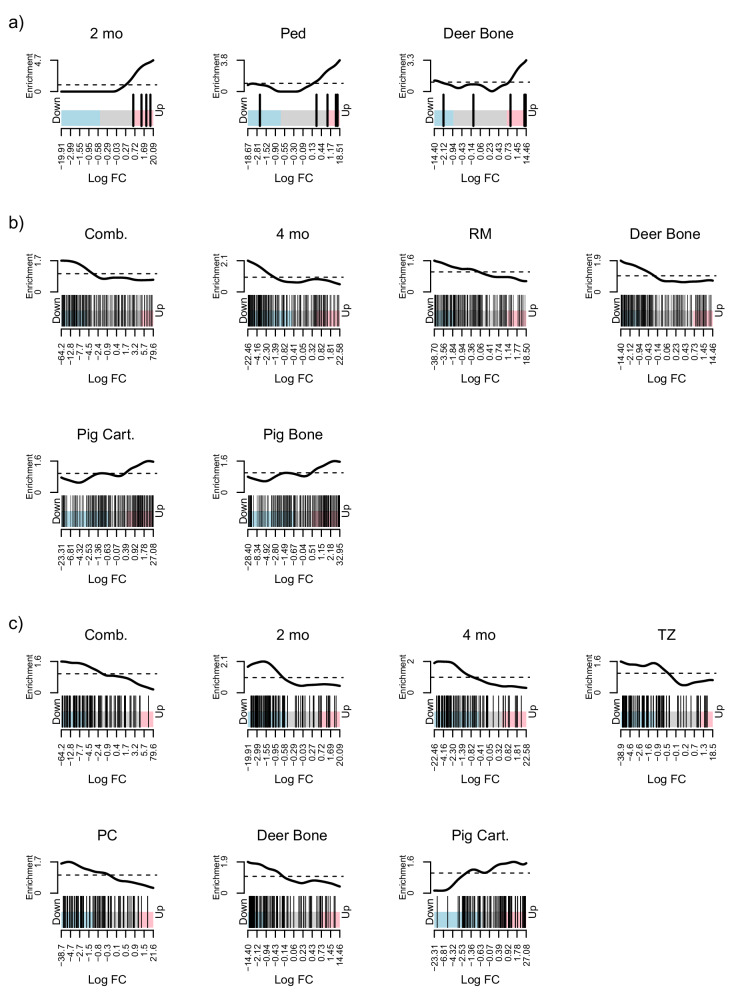
Highly ranked gene set barcode plots. Barcode plots of highly ranked gene sets with homologous expression patterns in cattle or deer tissues relative to pig tissues (either genes that tended to be expressed in a different direction or were not ranked). Each barcode plot represents the log fold changes (log FC) of genes in the set as vertical bars, regardless of significant differential expression (colored regions represent the range of significant differential expression log fold changes for each tissue). Lines above each barcode show local enrichment of these genes and their neighbors to represent the expression trends for genes in the set. **a** The gene set “Regulation of mesodermal cell fate specification” (GO:0042661) was significantly ranked and overexpressed in three tissues, including a cattle horn bud and deer antler tissue; this gene set was unranked in any of the pig tissues. **b** The Molecular Signatures Database (MSigDB) Hallmark gene set “Complement,” representing genes that are expressed as part of the complement system of innate immune response. Cattle and deer cranial appendage tissues and deer bone tended to underexpress the genes in this set, while pig cartilage and bone overexpressed them. C The MSigDB Hallmark gene set “IL6 JAK STAT3 signaling” was significantly ranked and underexpressed in all three cattle horn bud comparisons, two isolated deer antler tissues, and deer bone, but overexpressed in pig cartilage. Figure panels display combined cattle horn buds (Comb.), 2-month-old cattle horn bud (2 mo), 4-month-old cattle horn bud (4 mo), bulk deer antler pedicle (Ped), isolated deer antler transition zone (TZ), isolated deer antler pre-cartilage (PC), isolated deer antler reserve mesenchyme (RM), and control cartilage (Cart.), skin (Skin), and bone (Bone) unrelated to cranial appendages.

To understand functional enrichment in our DE results, we performed competitive gene set rankings of Molecular Signatures Database (MSigDB) Hallmark biological process genes^28^ and pathways related to bone and limb development. The 2-month-old cattle horn bud shared more significantly ranked gene sets with cattle skin and other bulk tissues than the 4-month-old horn bud did (a table of highly ranked gene sets for each horn bud analysis compared to the other test tissues is in supplementary data 4). We focused on highly ranked gene sets that distinguish cranial appendage samples from cattle or deer skin control tissues in homology tests. Three gene sets fit our homology criteria and were not significant or were enriched in the opposite direction in outgroup pig tissues; however, they were shared with control deer bone (i.e., bone unrelated to cranial appendages). These gene sets represent a developmental process (“Regulation of mesodermal cell fate specification,” GO:0042661), which was significantly ranked and overexpressed in 2-month-old horn buds, bulk antler pedicles, and control deer bone (Fig. 4a), and two Hallmark gene sets related to immune responses (“Complement” and “IL6 JAK STAT3 signaling”), which were highly ranked and underexpressed among multiple horn bud, isolated antler tissues, and control deer bone (Fig. 4b, c). As expected due to ossification differences, antler tissues had highly ranked gene sets related to chondrocytes and endochondral bone formation, while horn buds did not.

Because gene sets related to bone development may be shared among all bones regardless of whether they are cranial appendages, we also identified regulatory genes associated with significant gene sets and assessed whether they also were significantly DE in our test tissues. Among the regulatory genes identified for these gene sets, only *ALX1* fit our homology criteria and was overexpressed in 2- and 4-month-old cattle horn buds and in deer antler pedicles.

### Homology in tissue-specific genes

We produced two lists of tissue-specific genes using the tau metric (*τ*). Our strict specificity list (*τ* > 0.9) includes nine genes that meet our homology criteria (Table 1). Five genes were specific to the 2-month-old horn bud tissue, which shared the most specific genes with isolated antler reserve mesenchyme. These five genes represent a variety of functions and pathways, from cell adhesion to Wnt signaling. Among the highly specific genes was *RXFP2*, a gene previously implicated in horn development^6,23,29^. Four genes were specific to 4-month-old horn bud and shared with the bulk antler pedicle sample, also representing diverse pathways and processes.

**Table 1:** Homologous genes based on the strict specificity metric (*τ* > 0.9)

	Cattle		Deer						
	2 mo	4 mo	Ped	Ant	MC	CA	TZ	PC	RM
*NCAM2*	•								•
*SCG2*	•					•			
*DMP1*	•				•				
*RXFP2*	•								•
*SFRP2*	•								•
*GAD2*		•			•				
*TMEM211*		•	•						
*TMIE*		•				•			
*TYR*		•	•						

Genes that were specific to a single tissue within each taxon’s gene counts based on a strict threshold for the tau measure of tissue specificity (*τ* > 0.9) represented with •. Skin, cartilage, and bone unrelated to cranial appendages are not included here, because genes could only be specific to a single tissue under this metric. *2 mo* 2-month-old cattle horn bud, *4 mo* 4-month-old cattle horn bud, *Ped* bulk deer antler pedicle, *Ant* bulk deer antler tip, *MC* isolated deer antler mineralized cartilage, *CA* isolated deer antler cartilage, *TZ* isolated deer antler transition zone, *PC* isolated deer antler pre-cartilage, *RM* isolated deer antler reserve mesenchyme.

Among genes in our relaxed specificity list (*τ* > 0.75), 11 were specific to the 2-month-old horn bud tissue and one or more antler tissues (Table 2): the bulk pedicle sample shared the greatest number with 2-month-old horn bud (seven of these 11 genes), and the bulk antler tip, isolated antler transition zone, pre-cartilage, and reserve mesenchyme each shared six of these genes. Four-month-old horn buds had 23 genes on this list, sharing the highest number with the isolated antler transition zone (13 genes) and the next highest number with isolated antler pre-cartilage (9 genes). Seven genes were specific to both 2- and 4-month-old horn bud tissues and mapped as homologous, bringing the total of genes representing these tissues to 18 and 30, respectively. The relaxed specificity gene list thus had 12 total genes shared between 2-month-old cattle horn buds and bulk deer antler tips (a proportion of 0.67, Table 2), followed by bulk antler pedicle and isolated antler reserve mesenchyme each sharing 11 genes (0.61). Four-month-old cattle horn bud tissues shared 16 genes total with isolated deer antler transition zone (0.53) and 13 with the deer antler isolated pre-cartilage (0.43).

**Table 2:** Homologous genes based on the relaxed specificity metric (*τ* > 0.75)

	Cattle		Deer						
	2 mo	4 mo	Ped	Ant	MC	CA	TZ	PC	RM
*ASXL3*	•		•	•		•	•	•	•
*C1QTNF3*	•			•			•	•	•
*EPHA5*	•						•	•	•
*ESR2*	•		•	•					
*GABRA3*	•		•						
*IGF2*	•		•	•	•	•			•
*KAZALD1*	•		•		•				
*LGALS1*	•			•			•	•	•
*NALCN*	•					•	•	•	•
*PDZD2*	•		•		•				
*SERPINF1*	•		•	•	•	•	•	•	
*BNIP3*		•					•	•	
*DOLPP1*		•					•		
*EFCAB1*		•			•	•			
*EXOC3L2*		•			•	•	•		
*FAM167B*		•		•	•	•	•		
*FOSL1*		•		•			•	•	
*FRAS1*		•	•						
*GPI*		•					•	•	
*GPR1*		•			•		•	•	
*HSD17B2*		•		•					
*KCNA2*		•	•						
*LDHA*		•					•		
*LINGO1*		•				•			
*NSDHL*		•		•			•	•	•
*PCSK6*		•				•	•		
*PDLIM1*		•					•	•	•
*PKM*		•		•			•	•	•
*SFXN2*		•			•		•	•	
*SH3BP5*		•			•				
*SMPD5*		•	•		•				
*SMPDL3B*		•	•						
*WNT9B*		•				•			•
*YWHAH*		•				•		•	•
*CA12*	•	•		•		•	•		
*CHEK1*	•	•	•	•		•	•	•	•
*GARNL3*	•	•	•					•	•
*LIPG*	•	•		•					•
*ST18*	•	•		•	•	•			
*STMN4*	•	•	•	•	•			•	•
*TTK*	•	•	•	•		•	•	•	•
Prop. 2 mo			0.61	0.67	0.33	0.44	0.50	0.56	0.61
Prop. 4 mo			0.27	0.37	0.30	0.37	0.53	0.43	0.33

Genes with homologous tissue specificity based on a relaxed threshold for the tau measure of tissue specificity (*τ* > 0.75) represented with •. This relaxed threshold permitted identification of genes that would be excluded by the strict threshold (*τ* > 0.9) because they are highly expressed in more than one cranial appendage tissue. Genes that also were specific to cattle or deer skin and bone unrelated to cranial appendages under this metric were excluded from homology tests. Final two rows represent the proportion (Prop.) of homologous genes shared between 2-month-old cattle horn buds (of 18 total) and 4-month-old cattle horn buds (of 30 total) for each test deer antler tissue. *2 mo* 2-month-old cattle horn bud, *4 mo* 4-month-old cattle horn bud, *Ped* bulk deer antler pedicle, *Ant* bulk deer antler tip, *MC* isolated deer antler mineralized cartilage, *CA* isolated deer antler cartilage, *TZ* isolated deer antler transition zone, *PC* isolated deer antler pre-cartilage, *RM* isolated deer antler reserve mesenchyme.

### Gene clustering by self-organizing map (SOM)

**Fig. 5:**
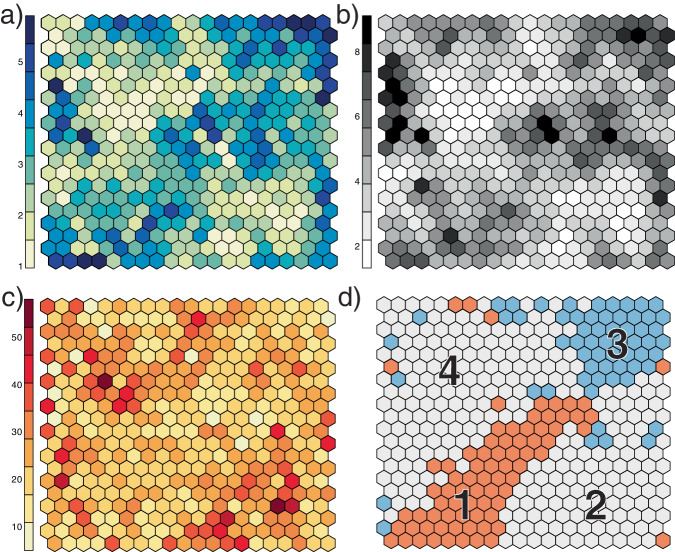
Self-organizing map summary figures. Self-organizing map (SOM) of log counts per million for 9433 genes across cattle, deer, and pig transcriptome analyses, showing mapping quality, distance, and clustering metrics. **a** heat scale is quality scores based on the average sum of squares distance of the count profile for each gene in the SOM unit to the “codebook” vector of the unit; lower scores (lighter colors) represent shorter distances between a gene’s expression profile and the codebook vector for the unit; **b** heat scale is average sum of squares distances of each SOM unit from its neighbors; lighter regions show units that are closer to their neighbors, and thus have genes with more similar gene expression patterns; **c** heat scale is the number of genes mapped to each SOM unit; this metric is expected to be similar between units across the map, and most units on our map had 20–30 genes. **d** The map showing the four k-medoid clusters of SOM units. Genes in clusters 1 and 3 exhibited patterns that support cranial appendage homology: Cluster 1 genes tended to have average log counts per million higher in cattle (Bovidae) and deer (Cervidae) cranial appendage tissues than in pig outgroup tissues, and genes in Cluster 3 tended to have lower average counts in cranial appendage tissues than in pig outgroup tissues. The contribution to the SOM by each tissue and taxon is in Fig. 6.

**Fig. 6:**
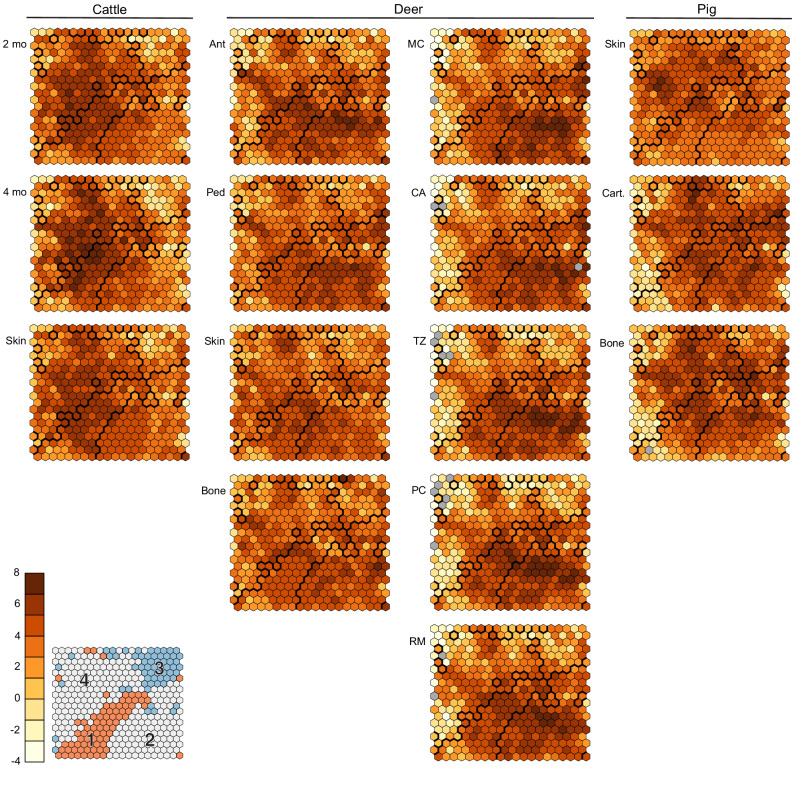
Tissue contributions to the self-organizing map. Each tissue’s contribution to the overall self-organizing map (SOM), showing the average log counts per million for genes mapping to each SOM unit. Bold lines mark the boundaries of k-medoid clusters, of which clusters 1 and 3 (see inset from Fig. 5D, where numbers correspond to cluster numbers, and Table 3) show patterns that differentiate cranial appendage (cattle horn and deer antler) tissues from outgroup pig skin, cartilage, and bone. Heat scale is log counts per million; gray units had zero counts per million for that tissue and species. Figure panels display 2-month-old cattle horn bud (2 mo), 4-month-old cattle horn bud (4 mo), bulk deer antler tip (Ant), bulk deer antler pedicle (Ped), isolated deer antler mineralized cartilage (MC), isolated deer antler cartilage (CA), isolated deer antler transition zone (TZ), isolated deer antler pre-cartilage (PC), isolated deer antler reserve mesenchyme (RM), and control cartilage (Cart), skin (Skin), and bone (Bone) unrelated to cranial appendages.

We found support for four k-medoid clusters in a 20-by-20-unit SOM of gene log counts per million (LCPM) (Fig. 5). Clusters 1 and 3 contained genes that generally had higher (cluster 1) or lower (cluster 3) mean LCPM in cranial appendages than in the outgroup pig samples (Table 3). In Cluster 1, however, both isolated antler mineralized cartilage and isolated antler cartilage had lower mean counts than pig cartilage. Deer antler tissues also did not always have higher counts in cluster 1 or lower counts in cluster 3 than the deer skin and bone controls. The SOM showed both taxon-specific and cranial appendage-specific regions of higher count genes across test tissues (Fig. 6). Genes with high counts in cattle samples mapped to clusters 1 and 4. Both cattle horn bud samples had their highest-count SOM units in cluster 4; the older horn bud sample had more of these high-count clusters across clusters 1 and 4. Genes with higher counts in deer antler samples mapped to clusters 1 and 2, with a strong peak in cluster 2 that appears in both the bulk and isolated antler samples to varying extents. Finally, the outgroup pig samples had less defined peaks; their highest-count units were widely distributed in clusters 2, 3, and 4. Regions with gene counts that may best support homology include the higher count region of cluster 1 (toward the center of the map) and the lower count regions of cluster 3.

**Table 3:** Mean counts by tissue of genes in each k-medoid cluster of SOM units

	Cattle			Deer									Pig		
Cluster	2 mo	4 mo	Skin	Ped	Ant	CA	MC	PC	RM	TZ	Skin	Bone	Skin	Cart.	Bone
1	5.08	5.36	4.99	4.61	4.71	4.52	4.48	4.86	4.85	4.65	4.70	4.57	3.79	4.53	4.25
2	3.81	3.67	4.15	5.09	4.91	5.38	5.57	5.29	5.24	5.39	4.48	4.69	3.60	4.89	4.35
3	2.77	1.98	3.06	3.41	2.96	3.14	3.16	2.13	2.84	2.39	3.11	3.35	3.59	4.39	4.45
4	4.86	4.89	4.74	3.53	3.43	2.83	2.63	2.73	2.84	2.72	3.91	3.75	4.34	4.11	4.30

Mean log counts per million of the genes that mapped to each of the four k-medoid clusters of SOM (self-organizing map) units, excluding genes with raw counts of zero, which had log counts per million of −21 due to the small integer added to each count to avoid taking the log of zero by *cpmByGroup*^57^. We determined the number of k-medoid clusters based on the Tibshirani et al.^70^ statistic. Genes in clusters 1 and 3 have mean counts that tended to be higher or lower (respectively) than those in control samples of pig skin, cartilage, and bone, supporting homology of cranial appendages in cattle horn buds and deer antlers. *2 mo* 2-month-old cattle horn bud, *4 mo* 4-month-old cattle horn bud, *Ped* bulk deer antler pedicle, *Ant* bulk deer antler tip, *MC* isolated deer antler mineralized cartilage, *CA* isolated deer antler cartilage, *TZ* isolated deer antler transition zone, *PC* isolated deer antler pre-cartilage, *RM* isolated deer antler reserve mesenchyme, *Cart.* cartilage unrelated to cranial appendages.

## Discussion

We tested the homology of horns and antlers based on the genes they express during development. We hypothesized that juvenile cattle horn buds are homologous to deer antler pedicle tissues, or the homologous core concept. Because the bony parts of cattle horns and deer antler pedicles both form through postnatal intramembranous ossification (entirely and in part, respectively), we predicted that our 2-month-old horn bud sample would share more homologously expressed genes with the antler pedicle than with antler tissues that form at later stages of development. Although development itself can evolve^25^, conserved gene expression patterns assessed within a phylogenetic context could reinforce the shared position on the frontal bone and intramembranous growth stages as evidence for horn and antler homology.

We discovered multiple homologously significantly DE genes in both cattle horns and deer antlers relative to skin, cartilage, and bone from our pig outgroup, supporting a shared, derived origin for these cranial appendages. Because horn buds at these stages are essentially skin (dermis and hypodermis) with additional capacity for inducing bone development and outgrowth^7^, the 2- and 4-month-old cattle horn buds both shared considerable expression patterns with their control skin samples. Likewise, the genes and pathways of bone formation are largely conserved across vertebrates^30,31^, so some gene expression patterns should overlap between cranial appendages and other bones. The timing and gene expression that determines where bone forms, however, can vary considerably^32^, thus conserved gene expression patterns that differentiated cranial appendages from other bone and outgroup tissues in our analyses may better indicate homology than overall gene expression similarity between any two tissues.

The combined age and 4-month-old cattle horn buds shared the greatest number of homologously expressed genes with isolated deer antler pre-cartilage; 2-month-old cattle horn buds shared the most homologously expressed genes with the bulk deer antler pedicle sample, and shared the second most with isolated antler pre-cartilage. Although we predicted the homologous core hypothesis would result in greater similarity between younger horn bud samples and younger antler tissues, this pattern may instead reflect homology through an early generalized signal that increases in specificity as cranial appendages develop. As a cross-section of constituent tissues in the antler pedicle, the bulk antler pedicle sample, in effect, averages expression across different tissue layers into a generalized signal. The isolated antler pre-cartilage, in contrast, shows distinct expression signals for a specific tissue. Expression similarity between cattle horn buds and deer antler tissues could refine our understanding of when cranial appendage development may diverge: Two-month-old horn buds still express a more generalized, bulk-tissue signal, while 4-month-old horn buds exhibit greater similarity to more specialized, isolated tissues. Horns do not grow by endochondral ossification, nor are cartilage precursors found in horns^1,3^; homologous gene expression patterns shared between cattle horn buds at any stage and isolated deer antler pre-cartilage thus may represent homologously conserved signals for initial bony outgrowth in both horns and antlers, rather than signals for cartilage formation.

Of particular interest for cranial appendage development are the expression of *TNRC6A* and *MYOC*. Two- and 4-month-old cattle horn buds, isolated deer antler mineralized cartilage, transition zone, and pre-cartilage all underexpressed *TNRC6A*, but non-antler deer bone samples overexpressed this gene. TNRC6A is a protein that helps micro-RNA silence genes^33^. Although the exact genes targeted by TNRC6A-mediated silencing in the non-antler deer bone are unclear, the underexpression of *TNRC6A* in cranial appendages may represent important regulatory differences from normal bone. For example, dysregulation of tumor-suppressing genes by micro-RNAs is prevalent in some cancers^34–36^. *TNRC6A* underexpression in the rapidly growing antler tissues could plausibly represent a mechanism that promotes their cancer-like growth rate or the stem-cell-like behaviors of antler-producing tissues. *MYOC* was homologously underexpressed by 4-month-old cattle horn buds and bulk deer antler pedicle but overexpressed in non-antler control deer bone. *MYOC* expression promotes the differentiation of mesenchymal stem cells into osteoblasts, especially through the MAPK14 (p38) pathway^37,38^. Knockout experiments in mice have shown that without *MAPK14* expression, bone density decreases^38^, and without *MYOC* expression, bone remodeling and osteoblast differentiation decrease^37^. *MAPK14* is homologously underexpressed in our 2-month-old cattle horn bud sample and the bulk deer antler pedicle, but it is not significantly expressed in either direction in any other tissue (see supplementary data 2). Future mechanistic studies of these genes during cranial appendage formation could provide additional evidence of regulatory expression changes that further support horn and antler homology.

Among the competitive gene set results, no gene sets distinguished cattle horn buds from all control tissues. The three highly ranked gene sets that mapped as homologous for horn and antler tissues, but also were shared with control deer bone, may reinforce the pattern of more specified expression patterns in the older horn bud sample: 2-month-old cattle horn buds shared a pathway with bulk deer antler pedicles and isolated antler tissues, while 4-month-old cattle horn buds shared pathways only with isolated deer antler tissues. These three gene sets may relate to bone formation and homeostasis^39–41^, demonstrating evidence for bone formation signals in cattle horn buds even at early juvenile stages. Although chondrocyte and endochondral ossification pathways were significant only in deer antlers, 2-month-old cattle horn buds did significantly express cartilage marker genes (e.g., *SOX9* and *ACAN*). *SOX9*, however, also may be a marker for neural crest cells^42^, and two- and 4-month-old cattle horn buds overexpressed multiple cranial neural crest cell markers: *ALX1*, *CRABP1*, *DLX1*, *DLX2*, *ID*, *SOX10, TFAP2A, TFAP2B*, and *TFAP2C* ^43,44^. Although the embryonic cells that give rise to horns are yet unknown, our results lend additional support to previous molecular studies suggesting cranial neural crest cells are responsible for both horn and antler formation^6^, which could indicate cranial appendage homology through even earlier developmental stages.

Further evidence for shared cranial neural crest origins of horns and antlers comes from regulatory genes associated with significantly ranked gene sets. Of the 11 genes in the “limb-bud formation” gene set, at least one cattle horn bud DE *SOX9*, *WNT3*, and *FGF10*, bulk deer antler pedicles DE *SOX9* and *WNT3*, and isolated deer antler transition zone and pre-cartilage DE *SOX9*. We do not think that cranial appendages are expressing a limb-bud signal; rather, the similarity likely relates to genes that pattern mesenchymal development in both the face (including the frontal bone) and limbs, in particular *ALX* family genes^45,46^. TRANSFAC v7.4^47^ identifies a binding motif within two kilobases before or after the transcription start site of *WNT3* for the cranial neural crest patterning gene *ALX1*. *ALX1* overexpression appears only in the two- and 4-month-old cattle horn buds and bulk deer antler pedicle samples in our analyses. *ALX1* sequence and expression differences in cranial neural crest cells can cause morphological variation within and between species^48–50^. Overexpression of *ALX1* in cattle horn buds and deer antler pedicle samples thus may represent a conserved mechanism for patterning cranial neural crest cells originally inherited from a common ancestral cranial outgrowth that now patterns these divergent horns and antlers.

Our analyses of tissue specificity of gene expression (*τ*) produced homologous patterns that better matched our predictions of greater similarity among the younger tissues. Two-month-old horn buds shared the highest proportions of relaxed specific genes with bulk antler tip (0.67, Table 2), bulk antler pedicle (0.61), and isolated antler reserve mesenchyme (0.61). Shared proportions of these genes decreased with each relatively older developmental stage, so that 2-month-old horn buds shared the fewest genes with isolated antler mineralized cartilage. Four-month-old horn buds shared the greatest number of tissue-specific genes with the isolated deer antler transition zone (0.53), and the proportion of shared genes decreased from this peak with each successive older or younger antler developmental stage. This pattern among the isolated deer antler tissues precisely matches our prediction under the homologous core concept, with younger 2-month-old cattle horn bud corresponding to earlier isolated deer antler tissues and the older 4-month-old cattle horn bud corresponding to an older isolated deer antler tissue. Furthermore, 2-month-old horn buds shared their highest proportions of genes with bulk pedicle and antler samples, resembling the pattern in homologously DE genes. Two-month-old cattle horn buds also shared higher proportions of genes with each deer antler tissue in general than did 4-month-old horn buds, potentially representing decreased similarity between horns and antlers as family-specific cranial appendage expression patterns diverge.

Among the highly specific genes that optimized as homologous between horns and antlers was *RXFP2*, shared by 2-month-old horn buds and isolated antler reserve mesenchyme. Previous research suggests that *RXFP2* expression is required for cranial appendage formation, and that loss of function mutations to the gene result in the *polled* phenotype among cattle^23,29^. Pseudogenization of *RXFP2* shared by musk deer (Moschidae) and the antlerless deer *Hydropotes inermis* (Cervidae) also may support cranial appendage homology^6^. Although our data support cranial appendage homology, changes to *RXFP2* alone are not adequate evidence of homology. Perturbation of *RXFP2* expression in some sheep breeds does not affect horn formation^51^. In our analyses, *RXFP2* was not DE in any cattle tissue, yet it was overexpressed in all antler tissues and outgroup pig skin. Further research is needed into the function of this gene in cranial appendage formation to test whether it indicates homology.

SOM clustering is a phenetic method, grouping genes by the similarity of their count patterns across the test tissues. Because clustering on the SOM included all genes significantly DE in at least one tissue but not significantly DE in every tissue, the similarity of horn buds and antler tissues to skin, cartilage, and bone unrelated to cranial appendages could have dominated the mapping. Indeed, both bone and skin also express many of the genes linked to cranial appendages^6^. We nevertheless found two regions of our SOM where gene counts in horn and antler tissues differed from the bone, skin, and cartilage samples, supporting cranial appendage homology. In clusters 1 and 3, the cranial appendage samples generally had mean counts that were respectively higher and lower than the pig skin, cartilage, and bone tissues. That we can distinguish patterns shared between cranial appendage and not outgroup tissues even in this phenetic clustering method suggests meaningful differences between tissues, and offers further evidence of their homology.

Our efforts to understand cranial appendage homology through gene expression in this study were limited by age differences between the samples of the different species in our study. Including a variable for age in our linear models allowed us to account for these differences. As a result of our rigorous, conservative modeling, however, if a gene’s expression differs greatly by age in any of the cranial appendage tissues, it would not be significant in our model and thus could not be tested for homologous expression. Although using this model increased our confidence that these genes are DE in cranial appendage tissues, it likely reduced the number of genes available overall for homology assessment between horn buds and more mature tissues (e.g., bulk pedicles, isolated antler mineralized cartilage), potentially obscuring a pattern that better matched our predictions. Furthermore, measuring expression across different deer species, although similar to methods used in other cranial appendage homology studies^6^, may have limited the power of those results. Increasing the number of equivalent age classes and tissue replicates within each species and including additional bovid and cervid species, as well as species in Giraffidae and Antilocapridae with different cranial appendage types, will improve these tests in the future. Further studies will continue to clarify cranial appendage gene expression patterns shared between different species that were likely inherited from a ruminant common ancestor.

Overall, our results supported the homology of horns and antlers. We identified multiple genes with homologous expression patterns across horns and antlers, including several transcription factors that suggest regulatory differences which may help distinguish cranial appendage bone growth from that of unrelated bones (*TNRC6A*, *MYOC*, *MAP14K*). Our results also provided support for a cranial neural crest origin for both horns and antlers, which may indicate conserved embryonic origins for these cranial appendages as well. Homology is fundamentally a question of phylogenetic optimization and reconstruction of shared ancestral states; our results show, especially with expression of genes like *RXFP2*, that inclusion of an outgroup species is required to judge whether expression patterns are conserved. Although the fossil record of cranial appendages and their morphological differences in extant and extinct ruminant taxa have obscured inferences of cranial appendage homology, carefully planned comparative phylogenetic analyses sampling appropriate outgroup taxa and integrating novel sources of data will continue to reveal their evolutionary history.

## Materials and methods

### Cattle horn bud RNA sequencing

With our guidance, a veterinarian collected one tissue sample each from the horn-forming tissues of three 2-month-old and three 4-month-old male cattle calves prior to routine disbudding, a process that removes the tissues responsible for horn growth. We selected these ages to gather information on horn-forming tissues during the earliest stages of development prior to the formation of the horn’s keratin sheath, which might not be homologous under the homologous core concept, and greatly complicates non-lethal soft tissue sampling methods. Calves were given an analgesic (Xylazine), and their cornual nerves were desensitized with a local anesthetic (Lidocaine). The veterinarian extracted full-depth samples of horn-forming soft tissues using 3 mm sterile biopsy punches, resulting in approximately cylindrical 3 mm diameter by 3–5 mm long samples. This procedure was reviewed and approved by the American Museum of Natural History’s Institutional Animal Care and Use Committee prior to sampling. We used SNP genotyping to confirm that all animals were homozygous for the recessive horned allele; this ensured that the study animals had true horns and not scurs or other horn abnormalities.

Excised tissues were immediately placed in RNA*later* (Qiagen, Valencia, CA, USA) to preserve RNA quality. Total RNA was extracted from tissues following a phenol-chloroform and RNeasy mini kit protocol and then sent to the New York Genome Center for TruSeq cDNA library preparation and paired-end Illumina HiSeq sequencing. We assessed read quality with FastQC^52^, then trimmed reads to improve quality with Trimmomatic^53^. First, we trimmed 15 bases from the start of all reads to remove regions where nucleotide proportions were highly variable. Next, we applied a four-nucleotide long sliding window trim, truncating each read when the average quality score in the window fell below 25. Finally, all sequences shorter than 25 base pairs were removed.

### Compiling data for comparative analyses

**Fig. 7:**
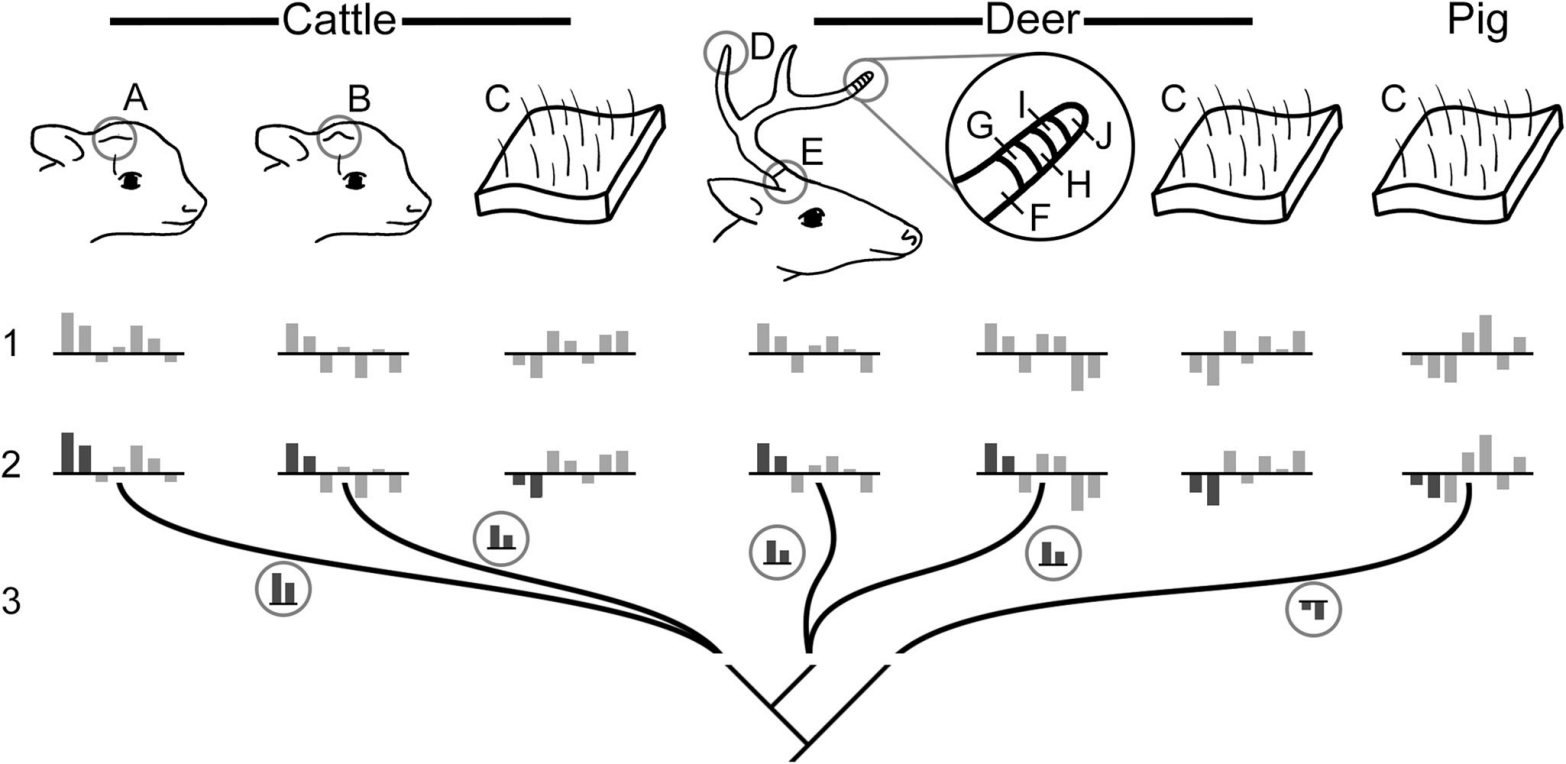
Schematic representation of tissue comparisons and analyses for transcriptomic homology assessment. Homology assessments followed three core steps: 1. Gene expression, tissue specificity, and gene set ranking determined for each test tissue. Bars above or below the line here represent hypothetical gene expression patterns (not based on actual data). 2. Results compared between cranial appendage tissues and within-species test controls (cattle skin or deer skin and bone) to identify significant genes that distinguish cranial appendages from these tissues, here highlighted in dark gray. 3. Homology evaluated by comparing distinct genes from step 2 (represented in circles) across species; genes that were similar in cattle horn and deer antler, but differed from outgroup pig skin, cartilage, and bone, were evidence of homology. A: 2-month-old cattle horn bud, B: 4-month-old cattle horn bud, C: test control tissues (cattle skin; deer skin and bone; pig skin, bone, and cartilage), D: bulk deer antler tip, E: bulk deer antler pedicle, F: isolated deer antler mineralized cartilage, G: isolated deer antler cartilage, H: isolated deer antler transition zone, I: isolated deer antler pre-cartilage, J: isolated deer antler reserve mesenchyme.

To identify DE genes in cattle horn bud tissues at 2- and 4-months-old relative to control tissues within the same species, we retrieved raw RNA sequence data for cattle skin from an area unrelated to cranial appendage growth, as well as a set of somatic control tissues—muscle, kidney, heart, liver, spleen, and lung—from GenBank (detailed information and accession numbers for each sample are in Table S1). Our goal was to establish differential gene expression for cranial appendage tissues and skin (test tissues) from an unrelated area against these somatic control tissues. When available for each taxon, we also included comparisons to cartilage and bone from anatomical regions unrelated to cranial appendages. Classically, horn-forming tissues are compared to other frontal bone skin^8,23^, or to skin from the horn region of polled, or hornless, animals^29^. These are sensible comparisons when studying a single species, but our use of a pig outgroup to establish homology required controls that could identify genes DE in other taxa without cranial appendages (i.e., pig skin). To maximize comparability of our horn buds to antler-forming tissues and non-cranial appendage-forming skin, cartilage, and bone in other species, especially given the lack of cranial appendage-forming skin in pigs, we measured differential gene expression in our cattle horn bud samples and published cattle skin sequences against the somatic control tissues (Fig. 7). We then compared the lists of significantly DE genes in cattle horn buds to those in cattle skin to identify which genes were DE only in the horn buds. We repeated this tissue comparison method for each deer antler sample against deer skin and bone.

For our antler pedicle and other antler tissue comparisons, we retrieved sequence data from GenBank for bulk antler pedicle soft tissues from adult sika deer (*Cervus nippon*), roe deer (*Capreolus capreolus*), and white-tailed deer *(Odocoileus virginianus*), and analyzed these together to achieve sufficient sample numbers for statistical analysis. We compiled sequence data for bulk antler tip tissues from adult sika and white-tailed deer. We also compiled data for growing antler tips sequenced as specific, isolated tissue layers (mineralized cartilage, cartilage, transition zone, pre-cartilage, reserve mesenchyme); the study authors followed a generalized protocol to dissect these tissues for genomic analysis^54,55^. These isolated tissue layers, although collected from adult animals of the same age (3 years old), represent an antler growth series that includes five sequential stages of mineralization in antler tip tissues, from initial reserve mesenchyme to final mineralized cartilage^54^. We use these comparisons rather than comparisons of equivalent developmental stages between cattle horns and deer antlers to explore potential heterochrony in expression patterns—if the two- or 4-month-old cattle horn buds share homologous expression patterns with more or less mineralized antler tissues, it may represent evolutionary changes in developmental timing between two homologous structures. For DE analyses, we also obtained sequence data from GenBank for deer skin and bone unrelated to antler growth, as well as for tissues from the same set of somatic control organs used for the cow horn bud analyses. To achieve at least triplicate representation of each antler tissue, samples from multiple species were analyzed together, hereafter referred to as deer, similar to strategies for analyzing antler transcriptomes in previous analyses^6^. For the phylogenetic outgroup comparison, we obtained sequences from GenBank for pig skin, cartilage, bone, and the same somatic control tissues collected for cattle and deer. We used data produced only by the Illumina short-read, high-throughput sequencing platform to ensure the greatest comparability to the horn bud samples sequenced for this study. All raw sequences retrieved from GenBank underwent the same quality control and trimming procedures as our newly sequenced cattle horn bud samples.

### Quantification

We aligned all cattle samples to the *Bos taurus* genome (UMD3.1.91), deer samples to the *Odocoileus virginianus* genome (Ovir.te_1.0), and pig samples to the *Sus scrofa* genome (Sscrofa11.1) using the *Rsubread* function *align*^56^. We counted each sequence that aligned to an exon and grouped those counts by gene name, using the default *featureCounts*^56^ settings, to produce a matrix of gene counts for each taxon. We analyzed each matrix separately and then compared results between species for homology analyses. For DE analyses, we filtered genes with fewer than 10 counts per million across any given sample within each tissue and at least 15 counts per million overall to remove genes unlikely to be expressed at biologically relevant levels using the *edgeR* function *filterByExpr*^57^. We then calculated TMM normalization factors for each matrix and estimated common, trended, and gene-wise dispersions using the “robust” setting to minimize outlier effects^57^. We also calculated LCPM for each gene within each taxon, including only genes with at least 10 counts in at least two-thirds of the replicates for each tissue using the *cpmByGroup* function in *edgeR*^57^.

### Differential expression analyses

We conducted separate DE analyses for our homology test tissues (the cranial appendage tissues as well as comparator control samples of cattle skin, deer skin, deer bone, pig skin, pig cartilage, and pig bone), relative to all somatic control tissues treated together as a single group within each taxon (Fig. 7). All DE analyses were performed in edgeR^57^. We fit quasi-likelihood negative binomial generalized linear models to each dataset, including the age, sex, and species of each sample as variables when relevant. We used two linear models for horn buds. In the first, we treated all horn bud samples together as a single tissue and included terms testing for the effects of the animals’ sex and age. We refer to this as the combined age model. Significant genes identified by this model are DE in cattle horn bud tissues regardless of age. Our second model treated the two ages of horn buds separately, retaining the sex variable but removing the age variable. Because no other cattle tissue sample was from two- or 4-month-old animals, a separate age variable in this model was colinear with the tissue type variable. We described results from this model based on the age of each horn bud sample. Linear models for deer and pig samples also included terms to account for sex and age of the samples. For the deer analysis, we included an additional term in the model for the species of the sample to account for potential between-species differences. We identified DE genes using Bayes quasi-likelihood F-tests^57^ for each test tissue relative to the somatic control samples, with a Benjamini-Hochberg correction for multiple testing (false discovery rate cutoff of 0.05)^58^.

### Competitive gene set ranking and regulatory overlaps

We also examined whether gene expression profiles in the different tissues may exhibit homologous functional enrichment using a competitive gene set test. We included the Broad Institute’s MSigDB reduced-redundancy Hallmark gene sets of biological processes^28^ in these analyses, as well as Biocarta pathways and biological process gene ontology (GO) terms related to spatial patterning, limb, and bone development also available through MSigDB. Results for all gene sets we tested within and between species are in supplementary data 4. Because this pathway information is stored in reference to human gene symbols, we first matched the genes to entrez gene IDs in the human genome annotation. For each test tissue, we determined whether genes within each gene set had average log fold changes significantly different from the average log fold change of genes not in the set, accounting for inter-gene correlations, with the edgeR function *camera*^57,59^. This competitive gene set ranking is a two-tailed test that also identifies whether genes in each set tend to be overexpressed or underexpressed^59^. Although not all genes in a set may be significantly overexpressed or underexpressed in the *camera* analysis, we use the terms “overexpressed” and “underexpressed” in describing these results to capture the general pattern of genes within each set.

Because there are limited pathways and genes involved in any bone development^60,61^, expression of genes in those pathways may only indicate deeply conserved mechanisms of bone formation rather than unique cranial appendage homology. We thus investigated potential regulatory genes of significantly ranked pathways. If cranial appendage-forming tissues rely on a different set of regulatory genes from other bone types, that could indicate distinct, shared evolutionary origins and homology. We identified possible regulatory genes for each significant gene set using the MSigDB “compute overlaps” function, which identified overlaps between genes in the set and MSigDB’s lists of regulatory gene targets^62,63^. If a gene set matched a regulatory gene target list with a false discovery rate-adjusted *q* value <0.05, we then checked our DE results for that regulatory gene to determine if it was DE in our test tissues.

### Gene specificity

Based on the LCPM matrix, we calculated *τ* (tau), a measure of the specificity of expression of a gene to a certain tissue^64,65^, for each gene within each taxon (i.e., cattle, deer, pigs). We classified genes with a *τ* ≥ 0.9 in a category of “strict specificity,” meaning the gene’s expression is specific to a single tissue. Because *τ* measures expression specificity in a single tissue, but our study has multiple cranial appendage tissues within the cattle and deer analyses, we also identified a set of genes with *τ* ≥ 0.75, which we referred to as “relaxed specificity.” The relaxed specificity list allowed us to examine genes where expression was high among multiple cranial appendage tissues within a taxon that otherwise would have been discarded under the more stringent threshold.

### Gene clustering by SOM

Finally, we clustered genes based on their expression patterns across the test tissues using a SOM^66,67^ implemented through the R package *kohonen*^68^. A SOM is a machine learning algorithm that clusters multivariate data into a two-dimensional map in which similar observations group in the same map unit or neighboring units; after training, each SOM unit has a “codebook” vector that has been updated to better reflect the observations mapping to it^66–68^. The number of neighboring units that are updated with each iteration of training decreases, until only one unit’s codebook vector updates with each match, at which point the clustering is analogous to k-means clustering^69^. We generated a 20-by-20-unit map of the LCPM matrix, scaled by each gene, for the 9,433 genes that were significantly DE in at least one test tissue across the three taxa. We used the sum of squares distances to determine the similarity between observations and the SOM units. Following SOM training, we clustered the SOM units into regions of greater similarity using a k-medoid clustering algorithm with four clusters, determining the best-supported number of clusters based on the Tibshirani et al.^70^ gap statistic. We calculated mean counts for each tissue type for the units within each cluster to assess whether clusters represented genes that were more similar among horn- and antler-forming tissues than among the other tissues. We excluded from these averages genes that had a raw count of zero, because their LCPM were -21 due to the small integer added to each count to avoid taking the log of zero by *cpmByGroup*^57^. The final SOM is included as supplementary data 5.

### Homology assessment

We assessed cranial appendage homology in each set of analyses (DE, gene set ranking, tissue specificity, SOM). In DE, gene set ranking, and tissue specificity homology assessments, we examined only genes or gene sets that were significantly DE, highly ranked, or specific in cranial appendage tissues but not in cattle skin, deer skin, or deer bone, unless the direction of expression differed. These steps focused our homology testing on genes that distinguish the soft tissues responsible for horn and antler growth from within-species test tissues that are not involved in cranial appendage formation. Furthermore, by analyzing the cattle or deer skin separately, rather than including them in the somatic control tissues for horn or antler DE analyses, we ensured that the comparison of horn and antler tissues to the pig skin outgroup is based on genes DE relative to tissues from the same set of organs. We considered any of these genes or gene sets that were expressed in a different direction, not statistically significant, or not specific in the pig skin, cartilage, and bone as a homologous pattern for horns and antlers. In the SOM, we did not exclude genes that were DE in non-cranial appendage tissues from the mapping, instead comparing average counts for each tissue in each unit cluster to assess homologous patterns.

### Statistics and reproducibility

Statistical analyses of DE, gene set ranking, and SOM clustering were conducted in R using standard workflows described in the supporting materials for each package. Tau (*τ*) calculations followed the code provided in Kryuchkova–Mostacci and Robinson-Rechavi^65^. Each tissue had at least three biological replicates (i.e., samples came from different animals), and *p* values were corrected for multiple testing with the Benjamini-Hochberg correction^58^. All RNA sequencing datasets are available through public repositories to support reproducibility.

### Reporting summary

Further information on research design is available in the Nature Portfolio Reporting Summary linked to this article.

### Supplementary information

Supplementary Information

Description of Additional Supplementary Materials

Supplementary Data 1

Supplementary Data 2

Supplementary Data 3

Supplementary Data 4

Supplementary Data 5

Reporting Summary

## Data Availability

Raw transcriptome sequences for 2-month-old and 4-month-old cattle horn buds newly sequenced for this study are available under GenBank Project Number PRJNA1088650. GenBank Accession numbers and citations for all literature data are in the supplementary information (Table S1).
