# Publisher Correction: Gene expression supports a single origin of horns and antlers in hoofed mammals

**DOI:** 10.1038/s42003-024-06425-w

**Published:** 2024-06-14

**Authors:** Zachary T. Calamari, John J. Flynn

**Affiliations:** 1 Division of Paleontology, American Museum of Natural History, Central Park West at 79th Street, New York, NY 10024 USA; 2 Richard Gilder Graduate School, American Museum of Natural History, Central Park West at 79th Street, New York, NY 10024 USA; 3 Present Address: Department of Natural Sciences, Baruch College, City University of New York, 17 Lexington Avenue, Box A-920, New York, NY 10010 USA

**Keywords:** Evolutionary genetics, Gene expression, Evolutionary developmental biology

Correction to: *Communications Biology* 10.1038/s42003-024-06134-4, published online 20 May 2024

The wrong Supplementary File (Supplementary Data 5) was originally published with this article; it has now been replaced with the correct file. The original article has been corrected.

