# Supplementary material for: Gene expression supports a single origin of horns and antlers in hoofed mammals
Source: Commun Biol. 2024 May 20;7:509. doi: 10.1038/s42003-024-06134-4 (PMC11106249; doi:10.1038/s42003-024-06134-4)
Supplement: Supplementary file 1 — Supplementary Information [file 42003_2024_6134_MOESM1_ESM.pdf]

Gene expression supports a single origin of horns and antlers in hoofed mammals

SUPPLEMENTARY INFORMATION

Zachary T. Calamari[1,2,*], John J. Flynn[2,3]

[1]Department of Natural Sciences, Baruch College, City University of New York, 17 Lexington Avenue, Box A-920, New York, NY 10010, USA.

[2]Division of Paleontology, American Museum of Natural History, Central Park West at 79th Street, New York, NY 10024, USA.

[3]Richard Gilder Graduate School, American Museum of Natural History Central Park West at 79th Street, New York, NY 10024, USA.

*Corresponding author, zachary.calamari@baruch.cuny.edu

Supplementary Information (this document):
Table S1 –
GenBank accession numbers, attributes, and citations for all tissues examined in this study.

List of Supplementary Data Files:
Supplementary Data 1 – Table of significant differentially expressed genes for the combined horn bud age analysis, including only genes that were present in the deer and pig annotations (i.e., excluding unidentified transcripts from the cattle genome).

Supplementary Data 2 – Table of significant differentially expressed genes for the two- and four-month-old horn bud analyses. As with supplementary data 1, we include only genes that were present in the deer and pig annotations.

Supplementary Data 3 – Table of significantly differentially expressed genes that mapped as homologous. These genes are expressed in the same direction in horn and antler tissues and expressed in the opposite direction, or not significantly differentially expressed, in the pig samples.

Supplementary Data 4 – Table of gene sets used for competitive gene set ranking tests and results.

Supplementary Data 5 – File containing the final self-organizing map (SOM) used for clustering analyses in Rdata format. Access the SOM using the *kohonen* package (see main text for SOM training parameters) in R:

```
library(kohonen)
load("Supplementary_Data_5.Rdata")
#data is stored as object named "all_som9.3"
plot(all_som9.3, type = "quality")
```

Table S1 – GenBank accession numbers, attributes, and citations for all tissues examined in this study.

Taxon	Tissue	Accession	Age	Sex	Location	Reported Condition	*Species*	Citation
Cattle	BH3	SRR28362214	2 months	Male	Farm	Healthy	*Bos taurus*	Present study
	BH8	SRR28362213	2 months	Male	Farm	Healthy	*Bos taurus*	Present study
	BH12	SRR28362211	2 months	Male	Farm	Healthy	*Bos taurus*	Present study
	BH2	SRR28362215	4 months	Male	Farm	Healthy	*Bos taurus*	Present study
	BH9	SRR28362212	4 months	Male	Farm	Healthy	*Bos taurus*	Present study
	BH14	SRR28362210	4 months	Male	Farm	Healthy	*Bos taurus*	Present study
	Skin	SRR1449246	2 years	Female	Farm	Euthanized due to leg injury	*Bos taurus*	1
		SRR1449247	2 years	Female	Farm	Euthanized due to leg injury	*Bos taurus*	1
		SRR1449248	2 years	Female	Farm	Euthanized due to leg injury	*Bos taurus*	1
		SRR1449250	2 years	Female	Farm	Euthanized due to leg injury	*Bos taurus*	1
		SRR1449251	2 years	Female	Farm	Euthanized due to leg injury	*Bos taurus*	1
		SRR1449252	2 years	Female	Farm	Euthanized due to leg injury	*Bos taurus*	1
	Muscle	SRR1449281	2 years	Female	Farm	Euthanized due to leg injury	*Bos taurus*	1
		SRR1449282	2 years	Female	Farm	Euthanized due to leg injury	*Bos taurus*	1
		SRR1449283	2 years	Female	Farm	Euthanized due to leg injury	*Bos taurus*	1
		SRR12697124	14 months	Male	Research institute	Not reported	*Bos taurus*	2
		SRR12697125	14 months	Male	Research institute	Not reported	*Bos taurus*	2
	Lung	SRR1449269	2 years	Female	Farm	Euthanized due to leg injury	*Bos taurus*	1
		SRR1449271	2 years	Female	Farm	Euthanized due to leg injury	*Bos taurus*	1
		SRR1449272	2 years	Female	Farm	Euthanized due to leg injury	*Bos taurus*	1
		SRR12697118	14 months	Male	Research institute	Not reported	*Bos taurus*	2
		SRR12697119	14 months	Male	Research institute	Not reported	*Bos taurus*	2
	Kidney	SRR1449266	2 years	Female	Farm	Euthanized due to leg injury	*Bos taurus*	1
		SRR1449267	2 years	Female	Farm	Euthanized due to leg injury	*Bos taurus*	1
		SRR1449268	2 years	Female	Farm	Euthanized due to leg injury	*Bos taurus*	1
	Heart	SRR1449263	2 years	Female	Farm	Euthanized due to leg injury	*Bos taurus*	1
		SRR1449264	2 years	Female	Farm	Euthanized due to leg injury	*Bos taurus*	1
		SRR1449265	2 years	Female	Farm	Euthanized due to leg injury	*Bos taurus*	1
	Liver	SRR1449243	2 years	Female	Farm	Euthanized due to leg injury	*Bos taurus*	1
		SRR1449244	2 years	Female	Farm	Euthanized due to leg injury	*Bos taurus*	1
		SRR1449245	2 years	Female	Farm	Euthanized due to leg injury	*Bos taurus*	1

		SRR12697112	14 months	Male	Research institute	Not reported	*Bos taurus*	2
		SRR12697113	14 months	Male	Research institute	Not reported	*Bos taurus*	2
	Spleen	SRR1449287	2 years	Female	Farm	Euthanized due to leg injury	*Bos taurus*	1
		SRR1449288	2 years	Female	Farm	Euthanized due to leg injury	*Bos taurus*	1
		SRR1449289	2 years	Female	Farm	Euthanized due to leg injury	*Bos taurus*	1
		SRR12697130	14 months	Male	Research institute	Not reported	*Bos taurus*	2
		SRR12697131	14 months	Male	Research institute	Not reported	*Bos taurus*	2
Deer	Bulk antler pedicle	SRR4256033	4 years*	Male	Wild	Not reported	*Odocoileus virginianus*	Baylor College of Medicine
		SRR8002929	1 year	Male	Farm	Not reported	*Cervus nippon*	3
		SRR8002935	7 months	Male	Farm	Not reported	*Capreolus capreolus*	3
	Bulk antler tip	SRR4256026	4 years	Male	Wild	Not reported	*Odocoileus virginianus*	Baylor College of Medicine
		ERR047168	4 years	Male	Farm	Anaesthetized	*Cervus nippon*	4
		ERR051744	4 years	Male	Farm	Anaesthetized	*Cervus nippon*	4
	Isolated antler mineralized cartilage	SRR6202143	3 years	Male	Not reported	Healthy	*Cervus nippon*	5
		SRR6202146	3 years	Male	Not reported	Healthy	*Cervus nippon*	5
		SRR6202147	3 years	Male	Not reported	Healthy	*Cervus nippon*	5
	Isolated antler cartilage	SRR6202144	3 years	Male	Not reported	Healthy	*Cervus nippon*	5
		SRR6202145	3 years	Male	Not reported	Healthy	*Cervus nippon*	5
		SRR6202156	3 years	Male	Not reported	Healthy	*Cervus nippon*	5
	Isolated antler transition zone	SRR6202148	3 years	Male	Not reported	Healthy	*Cervus nippon*	5
		SRR6202149	3 years	Male	Not reported	Healthy	*Cervus nippon*	5
		SRR6202157	3 years	Male	Not reported	Healthy	*Cervus nippon*	5
	Isolated antler pre-cartilage	SRR6202150	3 years	Male	Not reported	Healthy	*Cervus nippon*	5
		SRR6202151	3 years	Male	Not reported	Healthy	*Cervus nippon*	5
		SRR6202152	3 years	Male	Not reported	Healthy	*Cervus nippon*	5
	Isolated antler reserve mesenchyme	SRR6202153	3 years	Male	Not reported	Healthy	*Cervus nippon*	5
		SRR6202154	3 years	Male	Not reported	Healthy	*Cervus nippon*	5
		SRR6202155	3 years	Male	Not reported	Healthy	*Cervus nippon*	5
	Skin	SRR8002920	1 year	Male	Farm	Not reported	*Cervus nippon*	3
		SRR8002940	7 months	Male	Farm	Not reported	*Capreolus capreolus*	3

		SRR5647658	Adult	Not reported	Zoo	Not reported	*Rangifer tarandus*	6
	Bone	SRR8002921	1 year	Male	Farm	Not reported	*Cervus nippon*	3
		SRR8002941	7 months	Male	Farm	Not reported	*Capreolus capreolus*	3
		SRR4256027	4 years	Male	Wild	Not reported	*Odocoileus virginianus*	Baylor College of Medicine
	Muscle	SRR8002943	1 year	Male	Farm	Not reported	*Cervus nippon*	3
		SRR10867782	1 year	Not reported	Not reported	Not reported	*Cervus nippon*	7
		SRR10867783	3 years	Not reported	Not reported	Not reported	*Cervus nippon*	7
		SRR8002925	7 months	Male	Farm	Not reported	*Capreolus capreolus*	3
	Lung	SRR8002944	1 year	Male	Farm	Not reported	*Cervus nippon*	3
		SRR8002922	7 months	Male	Farm	Not reported	*Capreolus capreolus*	3
		SRR7410925	3 years	Male	Zoo	Accidental death from fight	*Axis porcinus*	8
		SRR4256029	4 years	Male	Wild	Not reported	*Odocoileus virginianus*	Baylor College of Medicine
	Kidney	SRR8002945	1 year	Male	Farm	Not reported	*Cervus nippon*	3
		SRR8002947	7 months	Male	Farm	Not reported	*Capreolus capreolus*	3
		SRR7410926	3 years	Male	Zoo	Accidental death from fight	*Axis porcinus*	8
	Heart	SRR8002957	1 year	Male	Farm	Not reported	*Cervus nippon*	3
		SRR7410922	3 years	Male	Zoo	Accidental death from fight	*Axis porcinus*	8
		SRR8002946	7 months	Male	Farm	Not reported	*Capreolus capreolus*	3
	Liver	SRR4256025	4 years	Male	Wild	Not reported	*Odocoileus virginianus*	Baylor College of Medicine
		SRR4256028	4 years	Male	Wild	Not reported	*Odocoileus virginianus*	Baylor College of Medicine
		SRR8002962	1 year	Male	Farm	Not reported	*Cervus nippon*	3
		SRR8002923	7 months	Male	Farm	Not reported	*Capreolus capreolus*	3
		SRR7410923	3 years	Male	Zoo	Accidental death from fight	*Axis porcinus*	8
	Spleen	SRR8002942	1 year	Male	Farm	Not reported	*Cervus nippon*	3
		SRR8002924	7 months	Male	Farm	Not reported	*Capreolus capreolus*	3
		SRR7410924	3 years	Male	Zoo	Accidental death from fight	*Axis porcinus*	8
Pig	Skin	SRR8173548	4 months	Not reported	Research institute	Local anaesthetic	*Sus scrofa*	9
		SRR8173549	4 months	Not reported	Research institute	Local anaesthetic	*Sus scrofa*	9
		SRR8173552	4 months	Not reported	Research institute	Local anaesthetic	*Sus scrofa*	9
		SRR8173553	4 months	Not reported	Research institute	Local anaesthetic	*Sus scrofa*	9
		SRR8173554	4 months	Not reported	Research institute	Local anaesthetic	*Sus scrofa*	9
		SRR8173555	4 months	Not reported	Research institute	Local anaesthetic	*Sus scrofa*	9
	Cartilage	SRR8471338	28 days	Female	Production company	Not reported	*Sus scrofa*	10

	SRR8471339	28 days	Female	Production company	Not reported	*Sus scrofa*	10
	SRR8471340	28 days	Female	Production company	Not reported	*Sus scrofa*	10
Bone	ERR2983233	7-32 months	Not reported	Research institute	Healthy/wildtype	*Sus scrofa*	11
	ERR2983234	7-32 months	Not reported	Research institute	Healthy/wildtype	*Sus scrofa*	11
	ERR2983235	7-32 months	Not reported	Research institute	Healthy/wildtype	*Sus scrofa*	11
	ERR2983236	7-32 months	Not reported	Research institute	Healthy/wildtype	*Sus scrofa*	11
Muscle	SRR5190390	Adult	Female	Not reported	Not reported	*Sus scrofa*	12
	SRR5190391	Adult	Female	Not reported	Not reported	*Sus scrofa*	12
	SRR5190396	Adult	Female	Not reported	Not reported	*Sus scrofa*	12
	SRR5190410	Adult	Female	Not reported	Not reported	*Sus scrofa*	12
	SRR5190413	Adult	Female	Not reported	Not reported	*Sus scrofa*	12
	SRR5190416	Adult	Female	Not reported	Not reported	*Sus scrofa*	12
Lung	SRR5190393	Adult	Female	Not reported	Not reported	*Sus scrofa*	12
	SRR5190395	Adult	Female	Not reported	Not reported	*Sus scrofa*	12
	SRR5190398	Adult	Female	Not reported	Not reported	*Sus scrofa*	12
	SRR5190409	Adult	Female	Not reported	Not reported	*Sus scrofa*	12
	SRR5190412	Adult	Female	Not reported	Not reported	*Sus scrofa*	12
	SRR5190415	Adult	Female	Not reported	Not reported	*Sus scrofa*	12
Kidney	SRR5190447	Adult	Female	Not reported	Not reported	*Sus scrofa*	12
	SRR5190448	Adult	Female	Not reported	Not reported	*Sus scrofa*	12
	SRR5190449	Adult	Female	Not reported	Not reported	*Sus scrofa*	12
	SRR5190504	Adult	Female	Not reported	Not reported	*Sus scrofa*	12
	SRR5190505	Adult	Female	Not reported	Not reported	*Sus scrofa*	12
	SRR5190506	Adult	Female	Not reported	Not reported	*Sus scrofa*	12
Heart	SRR5190392	Adult	Female	Not reported	Not reported	*Sus scrofa*	12
	SRR5190394	Adult	Female	Not reported	Not reported	*Sus scrofa*	12
	SRR5190397	Adult	Female	Not reported	Not reported	*Sus scrofa*	12
	SRR5190408	Adult	Female	Not reported	Not reported	*Sus scrofa*	12
	SRR5190411	Adult	Female	Not reported	Not reported	*Sus scrofa*	12
	SRR5190414	Adult	Female	Not reported	Not reported	*Sus scrofa*	12
Liver	SRR5190465	Adult	Female	Not reported	Not reported	*Sus scrofa*	12
	SRR5190467	Adult	Female	Not reported	Not reported	*Sus scrofa*	12
	SRR5190469	Adult	Female	Not reported	Not reported	*Sus scrofa*	12

	SRR5190477	Adult	Female	Not reported	Not reported	*Sus scrofa*	12
	SRR5190479	Adult	Female	Not reported	Not reported	*Sus scrofa*	12
	SRR5190481	Adult	Female	Not reported	Not reported	*Sus scrofa*	12
Spleen	SRR5190466	Adult	Female	Not reported	Not reported	*Sus scrofa*	12
	SRR5190468	Adult	Female	Not reported	Not reported	*Sus scrofa*	12
	SRR5190470	Adult	Female	Not reported	Not reported	*Sus scrofa*	12
	SRR5190478	Adult	Female	Not reported	Not reported	*Sus scrofa*	12
	SRR5190480	Adult	Female	Not reported	Not reported	*Sus scrofa*	12
	SRR5190482	Adult	Female	Not reported	Not reported	*Sus scrofa*	12

1. Chamberlain, A. J. *et al.* Extensive variation between tissues in allele specific expression in an outbred mammal. *BMC Genomics* **16**, 993 (2015).
2. Kern, C. *et al.* Functional annotations of three domestic animal genomes provide vital resources for comparative and agricultural research. *Nature Communications* **12**, 1821 (2021).
3. Wang, Y. *et al.* Genetic basis of ruminant headgear and rapid antler regeneration. *Science* **364**, 6446 (2019).
4. Yao, B. *et al. De novo* characterization of the antler tip of Chinese sika deer transcriptome and analysis of gene expression related to rapid growth. *Molecular and cellular biochemistry* **364**, 93–100 (2012).
5. Ba, H., Wang, D., Yau, T. O., Shang, Y. & Li, C. Transcriptomic analysis of different tissue layers in antler growth center in sika deer (*Cervus nippon*). *BMC Genomics* **20**, 173 (2019).
6. Figuet, E., Ballenghien, M., Lartillot, N. & Galtier, N. *Reconstruction of body mass evolution in the Cetartiodactyla and mammals using phylogenomic data*. 139147 https://www.biorxiv.org/content/10.1101/139147v3 (2017) doi:10.1101/139147.
7. Jia, B. *et al.* Altered miRNA and mRNA expression in sika deer skeletal muscle with age. *Genes* **11**, 172 (2020).
8. Wang, W. *et al.* The sequence and *de novo* assembly of hog deer genome. *Scientific Data* **6**, 180305 (2019).
9. Xu, Q. *et al.* Transcriptomic analysis of coding genes and non-coding RNAs reveals complex regulatory networks underlying the black back and white belly coat phenotype in Chinese Wuzhishan pigs. *Genes* **10**, 201 (2019).
10. Feng, S. *et al.* Identification of mRNAs related to tibial cartilage development of Yorkshire piglets. *BioMed Research International* **2019**, e2365416 (2019).
11. Niu, G. *et al.* Porcine model elucidates function of p53 isoform in carcinogenesis and reveals novel *circTP53* RNA. *Oncogene* **40**, 1896–1908 (2021).
12. Tang, Q. *et al.* Comparative transcriptomics of 5 high-altitude vertebrates and their low-altitude relatives. *GigaScience* **6**, (2017).
