# Supplementary material for: Gene expression supports a single origin of horns and antlers in hoofed mammals
Source: Commun Biol. 2024 May 20;7:509. doi: 10.1038/s42003-024-06134-4 (PMC11106249; doi:10.1038/s42003-024-06134-4)
Supplement: Supplementary file 2 — Description of Additional Supplementary Materials [file 42003_2024_6134_MOESM2_ESM.docx]

**Description of Additional Supplementary Files**

**File name:** Supplementary Data 1

**Description:** Table of significant differentially expressed genes for the combined horn bud age analysis, including only genes that were present in the deer and pig annotations (i.e., excluding unidentified transcripts from the cattle genome). Supports heatmap figures

**File name:** Supplementary Data 2

**Description:** Table of significant differentially expressed genes for the two- and four-monthold horn bud analyses. As with supplementary data 1, we include only genes that were present in the deer and pig annotations. Supports heatmap figures.

**File name:** Supplementary Data 3

**Description:** Table of significantly differentially expressed genes that mapped as homologous. These genes are expressed in the same direction in horn and antler tissues and expressed in the opposite direction, or not significantly differentially expressed, in the pig samples. Supports heatmap and homologous proportion figure.

**File name:** Supplementary Data 4

**Description:** Table of gene sets used for competitive gene set ranking tests and results. Supports barcode figure of functional enrichment analysis.

**File name:** Supplementary Data 5

**Description:** File containing the final selforganizing map (SOM) used for clustering analyses in Rdata format. Supports SOM figures.
