# Supplementary material for: Gene expression supports a single origin of horns and antlers in hoofed mammals
Source: Commun Biol. 2024 May 20;7:509. doi: 10.1038/s42003-024-06134-4 (PMC11106249; doi:10.1038/s42003-024-06134-4)
Supplement: Supplementary file 8 — Reporting Summary [file 42003_2024_6134_MOESM8_ESM.pdf]

nature portfolio

Corresponding author(s): Zachary T. Calamari

Last updated by author(s): Mar 18, 2024

Reporting Summary

Nature Portfolio wishes to improve the reproducibility of the work that we publish. This form provides structure for consistency and transparency in reporting. For further information on Nature Portfolio policies, see our Editorial Policies and the Editorial Policy Checklist.

Statistics

For all statistical analyses, confirm that the following items are present in the figure legend, table legend, main text, or Methods section.

n/a	Confirmed	
☐	☒	The exact sample size (n) for each experimental group/condition, given as a discrete number and unit of measurement
☐	☒	A statement on whether measurements were taken from distinct samples or whether the same sample was measured repeatedly
☐	☒	The statistical test(s) used AND whether they are one- or two-sided<br>*Only common tests should be described solely by name; describe more complex techniques in the Methods section.*
☐	☒	A description of all covariates tested
☐	☒	A description of any assumptions or corrections, such as tests of normality and adjustment for multiple comparisons
☐	☒	A full description of the statistical parameters including central tendency (e.g. means) or other basic estimates (e.g. regression coefficient) AND variation (e.g. standard deviation) or associated estimates of uncertainty (e.g. confidence intervals)
☐	☒	For null hypothesis testing, the test statistic (e.g. F, t, r) with confidence intervals, effect sizes, degrees of freedom and P value noted<br>*Give P values as exact values whenever suitable.*
☒	☐	For Bayesian analysis, information on the choice of priors and Markov chain Monte Carlo settings
☒	☐	For hierarchical and complex designs, identification of the appropriate level for tests and full reporting of outcomes
☒	☐	Estimates of effect sizes (e.g. Cohen's d, Pearson's r), indicating how they were calculated

Our web collection on statistics for biologists contains articles on many of the points above.

Software and code

Policy information about availability of computer code

Data collection	No software was used beyond proprietary Illumina software for determining basepair sequence and de-multiplexing sequencing reads.
Data analysis	Data quality control: FastQC 0.11.9<br>Sequence trimming: Trimmomatic 0.36<br>Transcript alignment and quantification: Rsubread 3.11<br>Differential gene expression analyses, log counts per million calculations, gene set ranking: edgeR 3.20.9<br>Self-organizing map: kohonen 3.0.12<br>Gene set function overlaps ("compute overlaps"): PANTHER 17.0

For manuscripts utilizing custom algorithms or software that are central to the research but not yet described in published literature, software must be made available to editors and reviewers. We strongly encourage code deposition in a community repository (e.g. GitHub). See the Nature Portfolio guidelines for submitting code & software for further information.

Data

Policy information about availability of data

All manuscripts must include a data availability statement. This statement should provide the following information, where applicable:

- Accession codes, unique identifiers, or web links for publicly available datasets
- A description of any restrictions on data availability
- For clinical datasets or third party data, please ensure that the statement adheres to our policy

Raw transcriptome sequences for two-month-old and four-month-old cattle horn buds newly sequenced for this study are available under GenBank Project Number PRJNA1088650. GenBank Accession numbers and citations for all literature data are in the supplementary information (Table S1).

Research involving human participants, their data, or biological material

Policy information about studies with human participants or human data. See also policy information about sex, gender (identity/presentation), and sexual orientation and race, ethnicity and racism.

Reporting on sex and gender	N/A
Reporting on race, ethnicity, or other socially relevant groupings	N/A
Population characteristics	N/A
Recruitment	N/A
Ethics oversight	N/A

Note that full information on the approval of the study protocol must also be provided in the manuscript.

Field-specific reporting

Please select the one below that is the best fit for your research. If you are not sure, read the appropriate sections before making your selection.

☒ Life sciences ☐ Behavioural & social sciences ☐ Ecological, evolutionary & environmental sciences

For a reference copy of the document with all sections, see nature.com/documents/nr-reporting-summary-flat.pdf

Life sciences study design

All studies must disclose on these points even when the disclosure is negative.

Sample size	Three samples were collected for both ages of juvenile horn tissues (two-month-old and four-month-old). Triplicate sampling is accepted as an appropriate number of samples for differential expression analyses.
Data exclusions	Genes with fewer than 10 counts per million across 2/3 of the samples for that tissue were discarded before analysis, per edgeR author recommendations, because genes expressed at such low levels are less likely to be biologically relevant.
Replication	All statistical tests were performed multiple times. The self-organizing map training may vary slightly from run to run, thus the output map is included as supplemental data.
Randomization	Allocation of samples was based on age. RNA was extracted from all samples at the same time to reduce potential batch effects and ensure similar extraction conditions. Age, sex, and species (when analyses included multiple species for a tissue) were included as covariates in the linear models.
Blinding	RNA was extracted from tissue samples and sent for sequencing using tubes marked with numbers that corresponded to animal ID numbers and not ages. Age of animals for the horn tissue samples is a key variable in this study, and was not associated with the data until statistical analyses were performed.

Reporting for specific materials, systems and methods

We require information from authors about some types of materials, experimental systems and methods used in many studies. Here, indicate whether each material, system or method listed is relevant to your study. If you are not sure if a list item applies to your research, read the appropriate section before selecting a response.

Materials & experimental systems

n/a	Involved in the study
☒	☐ Antibodies
☒	☐ Eukaryotic cell lines
☒	☐ Palaeontology and archaeology
☐	☒ Animals and other organisms
☒	☐ Clinical data
☒	☐ Dual use research of concern
☒	☐ Plants

Methods

n/a	Involved in the study
☒	☐ ChIP-seq
☒	☐ Flow cytometry
☒	☐ MRI-based neuroimaging

Animals and other research organisms

Policy information about studies involving animals; ARRIVE guidelines recommended for reporting animal research, and Sex and Gender in Research

Laboratory animals	Horn bud samples were collected by a veterinarian from 6 individuals, all Bos taurus (domestic cattle). Three samples were from two-month-old calves, and three were from four-month-old calves.
Wild animals	N/A
Reporting on sex	Due to differences in expression of genes between males and females, sex of animal source for each tissue sample was included as a variable in the linear models to assess differential expression (if unknown, it was recorded as such). Information about sample sex is included in the supplementary information.
Field-collected samples	Samples were collected and placed directly into RNAlater, then shipped overnight to the American Museum of Natural History, where they were stored at -80 degrees C until processing.
Ethics oversight	All sampling methods were approved by the American Museum of Natural History's Institutional Animal Care and Use Committee.

Note that full information on the approval of the study protocol must also be provided in the manuscript.
